# Retinoic Acid Mediates Regulation of Network Formation by COUP-TFII and VE-Cadherin Expression by TGFβ Receptor Kinase in Breast Cancer Cells

**DOI:** 10.1371/journal.pone.0010023

**Published:** 2010-04-06

**Authors:** Priya Prahalad, Sivanesan Dakshanamurthy, Habtom Ressom, Stephen W. Byers

**Affiliations:** Lombardi Comprehensive Cancer Center, Department of Oncology, Georgetown University, Washington, D. C., United States of America; Leiden University, Netherlands

## Abstract

Tumor development, growth, and metastasis depend on the provision of an adequate vascular supply. This can be due to regulated angiogenesis, recruitment of circulating endothelial progenitors, and/or vascular transdifferentiation. Our previous studies showed that retinoic acid (RA) treatment converts a subset of breast cancer cells into cells with significant endothelial genotypic and phenotypic elements including marked induction of VE-cadherin, which was responsible for some but not all morphological changes. The present study demonstrates that of the endothelial-related genes induced by RA treatment, only a few were affected by knockdown of VE-cadherin, ruling it out as a regulator of the RA-induced endothelial genotypic switch. In contrast, knockdown of the RA-induced gene COUP-TFII prevented the formation of networks in Matrigel but had no effect on VE-cadherin induction or cell fusion. Two pan-kinase inhibitors markedly blocked RA-induced VE-cadherin expression and cell fusion. However, RA treatment resulted in a marked and broad reduction in tyrosine kinase activity. Several genes in the TGFβ signaling pathway were induced by RA, and specific inhibition of the TGFβ type I receptor blocked both RA-induced VE-cadherin expression and cell fusion. Together these data indicate a role for the TGFβ pathway and COUP-TFII in mediating the endothelial transdifferentiating properties of RA.

## Introduction

Tumor growth and metastasis are dependent upon the presence of an adequate vascular supply. A breast tumor that is unable to properly vascularize can grow no larger than 4 mm^3^ or spread, and it was traditionally thought that angiogenesis was the sole method by which tumor cells can acquire an adequate vasculature. As a tumor expands, central necrosis occurs due to hypoxia and nutrient deprivation[1] leading to the production of angiogenic factors that recruit blood vessels from neighboring vessels or progenitor cells[2]. However, clinical trials with angiogenesis inhibitors have been disappointing.

The phenomenon of vasculogenic mimicry is one potential mechanism for tumor resistance to angiogenesis inhibitors [3] and increased patient mortality [4]. Vasculogenic mimicry refers to the ability of highly aggressive tumor cells to form matrix-rich networks surrounding spheroidal clusters of tumor cells in the absence of tumor necrosis and angiogenesis [5]. Observational data indicates that these tumor cells may also be able to interact with endothelial cells and line channels that conduct blood into the tumor [6], [7]. This phenomenon has been observed in vivo in melanoma, prostate, ovarian, liver, breast cancers, astrocytomas, mesothelial sarcomas, and sarcomas, as well as in vitro in highly aggressive melanoma and bladder cancer cell lines [6], [7], [8], [9], [10], [11], [12], [13], [14], [15], [16], [17]. Tumor cells exhibiting vasculogenic mimicry can upregulate the expression of endothelial specific genes [5], [18]. While markers of vasculogenic mimicry are being identified, the mechanism regulating vasculogenic mimcry or the factors inducing the phenomenon are still unknown.

Previously, we have shown that treatment of SKBR-3 breast cancer cells with 9-*cis*-retinoic acid (RA) induces the expression of endothelial specific genes, including VE-cadherin [19]. When these cells are grown in Matrigel, they form network-like structures, and RA treated SKBR-3 cells are able to fuse with each other. Additionally, RA-treated SKBR-3 cells are able to interact with HUVEC cells in Matrigel to form mixed vessel networks. Two factors, the HMG box protein SOX9 and the ets-family member ER81, were necessary for the RA induced expression of VE-cadherin [19].

In the present study we eliminate VE-cadherin as a master regulator of the RA-induced endothelial gene upregulation by showing that few of the many endothelial-related genes are affected by knockdown of VE-cadherin. COUP-TFII is an orphan nuclear receptor that is induced by RA treatment and involved in venous differentiation[20], [21], [22]. We found that knockdown of COUP-TFII prevented the formation of networks in Matrigel but had no effect on VE-cadherin induction and subsequent cell fusion. Surprisingly, considering the important role of tyrosine kinases in angiogenesis and vascular development, tyrosine kinases are not important in RA-mediated vascular mimicry [23]. In fact, RA-treatment resulted in a marked and broad reduction in tyrosine kinase activity. However, several genes in the TGFβ signaling pathway were induced by RA, and specific inhibition of the TGFβ type I receptor blocked both RA-induced VE-cadherin expression and cell fusion. Together these data indicate a role for the TGFβ pathway and COUP-TFII in mediating the endothelial transdifferentiating properties of RA.

## Results

### VE-cadherin, COUP-TFII, and NRP1 are not master regulators of endothelial transdifferentiation

We have previously shown that SOX9 and ER81 expression are necessary but not sufficient for RA-induced endothelial transdifferentiation. Since VE-cadherin is important for both vasculogenesis and angiogenesis, we wanted to determine which RA induced genes were dependent upon VE-cadherin expression. In our previous study, we treated SKBR-3 cells with 10^−6^ M RA. We repeated the experiment using 10^−7^ M RA (ArrayExpress accession: E-MEXP-2417) and found a similar induction of RA induced endothelial specific genes (Table 1). Using Ingenuity Pathway Analysis, we determined that the genes regulated by RA treatment belonged to the tumor morphology pathway (Table S1), cardiovascular development (Table S2), and hematological and coagulation pathways (Table S3).

**Table 1:** Top 25 Endothelial Related Genes Regulated by RA.

Gene Symbol	Description	Fold Change (RA/Control)	p-value
CDH5	cadherin 5, type 2, VE-cadherin (vascular epithelium)	10	7.0E-07
TFPI2	tissue factor pathway inhibitor 2	6.34	7.0E-07
BDKRB2	bradykinin receptor B2	5	2.0E-05
EFNB2	ephrin-B2	5	3.0E-03
CP	ceruloplasmin (ferroxidase)	5	1.1E-03
SELE	selectin E (endothelial adhesion molecule 1)	5	3.6E-06
SELL	selectin L (lymphocyte adhesion molecule 1)	5	1.0E-06
ID1	inhibitor of DNA binding 1, dominant negative helix-loop-helix protein	3.33	9.2E-05
COUP-TFII	nuclear receptor subfamily 2, group F, member 2	3.33	3.4E-05
PLAU	plasminogen activator, urokinase	3.33	2.2E-03
COX1	prostaglandin E synthase	3.33	4.6E-06
CAV1	caveolin 1, caveolae protein, 22 kDa	2.5	6.0E-04
NRP1	neuropilin 1	2.5	2.0E-04
TGFBR2	transforming growth factor, beta receptor II (70/80 kDa)	2.5	3.4E-03
ANXA2	annexin A2	2	2.6E-04
BDKRB1	bradykinin receptor B1	2	1.3E-04
CAV2	caveolin 2	2	1.5E-02
EPAS1	endothelial PAS domain protein 1	2	3.2E-03
SERPINE1	serpin peptidase inhibitor, clade E (nexin, plasminogen activator inhibitor type 1), member 1	2	3.5E-03
TGFB2	transforming growth factor, beta 2	2	2.8E-03
EPHB4	EPH receptor B4	−1.5	7.5E-03
F12	coagulation factor XII (Hageman factor)	−1.5	4.1E-03
TGFBR1	transforming growth factor, beta receptor I (activin A receptor type II-like kinase, 53 kDa)	−1.9	1.6E-02
TGFB3	transforming growth factor, beta 3	−2.5	3.89E-05

We then used microarray analysis to compare the genes induced by RA in the presence and absence of VE-cadherin (ArrayExpress accession: E-MEXP-2418) to analyze which induced genes are regulated by VE-cadherin. We transfected SKBR-3 cells with control luciferase siRNA or VE-cadherin siRNA. After 8 hours, cells were treated with 10^−7^M RA. The cells were allowed to grow for an additional 48 hours before RNA was collected and processed for microarray analysis.

Analysis of the microarray data using BRB Array Tools and the RMA Methodology yields 208 unique genes that are differentially regulated as a result of VE-cadherin siRNA transfection. We found that 10 of the genes we had identified as being endothelial related in our original microarray were differentially regulated in the presence of VE-cadherin siRNA (Table 2). Of those, only 3 genes – VE-cadherin itself, HMGCS1, and KLF5 – were reduced (compared to RA and control siRNA) in the absence of VE-cadherin. As expected VE-cadherin was markedly reduced, HMGCS1 and KLF5 were modestly affected (1.59 and 1.3 fold respectively). Seven other endothelial related genes were further induced (albeit slightly; 1.2–1.6 fold) in the absence of VE-cadherin. These data suggests that VE-cadherin may regulate some endothelial-related genes, but it does not serve as a master regulator of endothelial transdifferentiation.

**Table 2:** Endothelial-Related Genes Regulated by RA and VE-cadherin.

Gene Symbol	Gene Name	Fold Change with RA + Luc siRNA	Fold Change with RA + VE-cadherin siRNA
CCL2	Chemokine Ligand 2	2 (p = 2.0E-02)	2 (p = 1.1E-04)
**CDH5**	**VE-cadherin**	**10** (p = 7.0E-07)	**−3.8** (p = 6.5E-06)
CTSS	Cathepsin S	5 (p = 1.7E-04)	1.6 (p = 4.1E-04)
**HMGCS1**	**3-hydroxy-3-methylglutaryl-Coenzyme A synthase 1**	**1.4** (p = 9.1E-03)	**−1.6** (p = 6.5E-03)
HMOX1	Heme oxygenase 1	1.7 (p = 3.7E-03)	1.3 (p = 1.0E-02)
IGFBP3	Insulin-like Growth Factor Binding Protein 3	2.5 (p = 1.7E-03)	1.4 (p = 1.4E-03)
**KLF5**	**Kruppel Like Factor 5**	**2** (p = 1.6E-03)	**−1.3** (p = 2.4E-02)
MGP	Matrix Gla Protein	10 (p = 2.9E-06)	1.2 (p = 2.9E-02)
PLAU	Plasminogen Activator, Urokinase	3.3 (p = 2.2E-03)	1.7 (p = 6.3E-04)
TGFB2	TGFβ2	2 (p = 2.8E-03)	1.5 (p = 1.6E-03)

**Figure 1:**
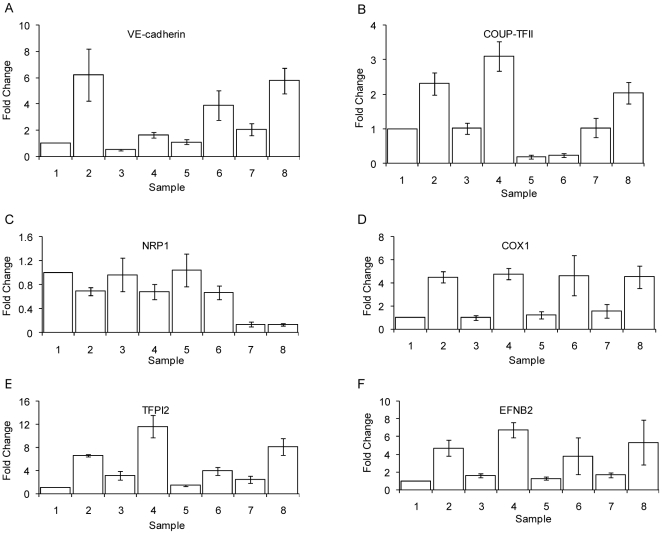
VE-cadherin, COUP-TFII, and NRP1 are not master regulators of retinoic acid mediated vasculogenic mimicry. Following siRNA transfection with luciferase control or siRNA targeting VE-cadherin, COUP-TFII, or NRP1, cells were treated with control ethanol or RA for 48 hours. qPCR analysis was performed to assess knockdown of VE-cadherin (a), COUP-TFII (b), NRP1 (c), COX1 (d), TFPI2 (e), and EFNB2 (f). Samples have been labeled as follows: 1 - Control, Luciferase siRNA; 2 - RA, Luciferase siRNA; 3 - Control, VE-cadherin siRNA; 4 - RA, VE-cadherin siRNA; 5 - Control, COUP-TFII siRNA; 6 - RA, COUP-TFII siRNA; 7 - Control, NRP1 siRNA; 8 - RA, NRP1 siRNA.

Since VE-cadherin is not responsible for the RA mediated induction of endothelial genes, we next examined the roles of COUP-TFII and NRP1 in regulating the expression of endothelial specific genes. COUP-TFII is an orphan nuclear receptor involved in venous differentiation, whereas NRP1 is a transmembrane glycoprotein that promotes artery formation. Both were significantly induced by RA [24], [25]. In addition to looking at the regulation of VE-cadherin, COUP-TFII, and NRP1, we used the expression of three other RA-regulated endothelial related genes – EfnB2, TFPI2, and COX1 – as markers of endothelial transdifferentiation. EfnB2, a ligand for the Eph receptor B4 (EphB4), is induced by NRP1 and is involved in artery formation[24], [25]. TFPI2, a serine protease inhibitor, is involved in the clotting cascade[26], whereas COX1 is a gene expressed by endothelial cells and involved in inflammation[27], [28]. To determine whether NRP1 or COUP-TFII can serve as regulators of endothelial transdifferentiation, we reduced the expression of these genes using siRNA, treated them with RA for 48 hours, and performed qPCR assays to determine the expression levels of various endothelial specific genes (Figure 1). qPCR analysis verifies the microarray result indicating that VE-cadherin may regulate the expression of TFPI2. Similar to VE-cadherin, neither COUP-TFII nor NRP1 regulate the expression of the other endothelial genes we measured. The 2.5 fold induction of NRP1 by RA observed by microarray analysis was not confirmed by qPCR further ruling out a role for this molecule. Taken together, these results indicate that VE-cadherin, COUP-TFII, and NRP1 do not regulate one another.

### Receptor tyrosine kinase activity is not important for RA-mediated VE-cadherin induction

We next examined the roles of kinases in regulating VE-cadherin expression and network formation. Activation of kinases by their ligands is important during most stages of angiogenesis and vasculogenesis. During vasculogenesis, binding of basic fibroblast growth factor to its receptor results in the differentiaton of mesodermal cells into hemangioblasts, precursors of both hematopoietic cells and endothelial cells[29], [30]. VEGF binding to its receptors, VEGFR-2 and VEGFR-1, is necessary for further differentiation of endothelial cells, endothelial cell-cell contacts, and vascular tube formation[31], [32], [33], [34]. Next, the binding of angiopoietin-1 (Ang1) to its receptor, Tie2, results in increased stabilization of the vascular wall via the recruitment of pericytes[35], [36], [37]. At this point, vasculogenesis is complete and angiogenesis begins to model a mature vascular system. VEGF works to loosen cell-contacts and breaks down the basement membrane so that endothelial cells can migrate and form sprouts[38]. Next, platelet derived growth factor (PDGF) binds to its receptor, PDGFβR, and recruits pericytes to strengthen the capillary wall[39]. TGF-β signaling via the TGFβ type I receptor has both pro-angiogenic and anti-angiogenic effects on proliferation and migration[40], [41].

**Figure 2:**
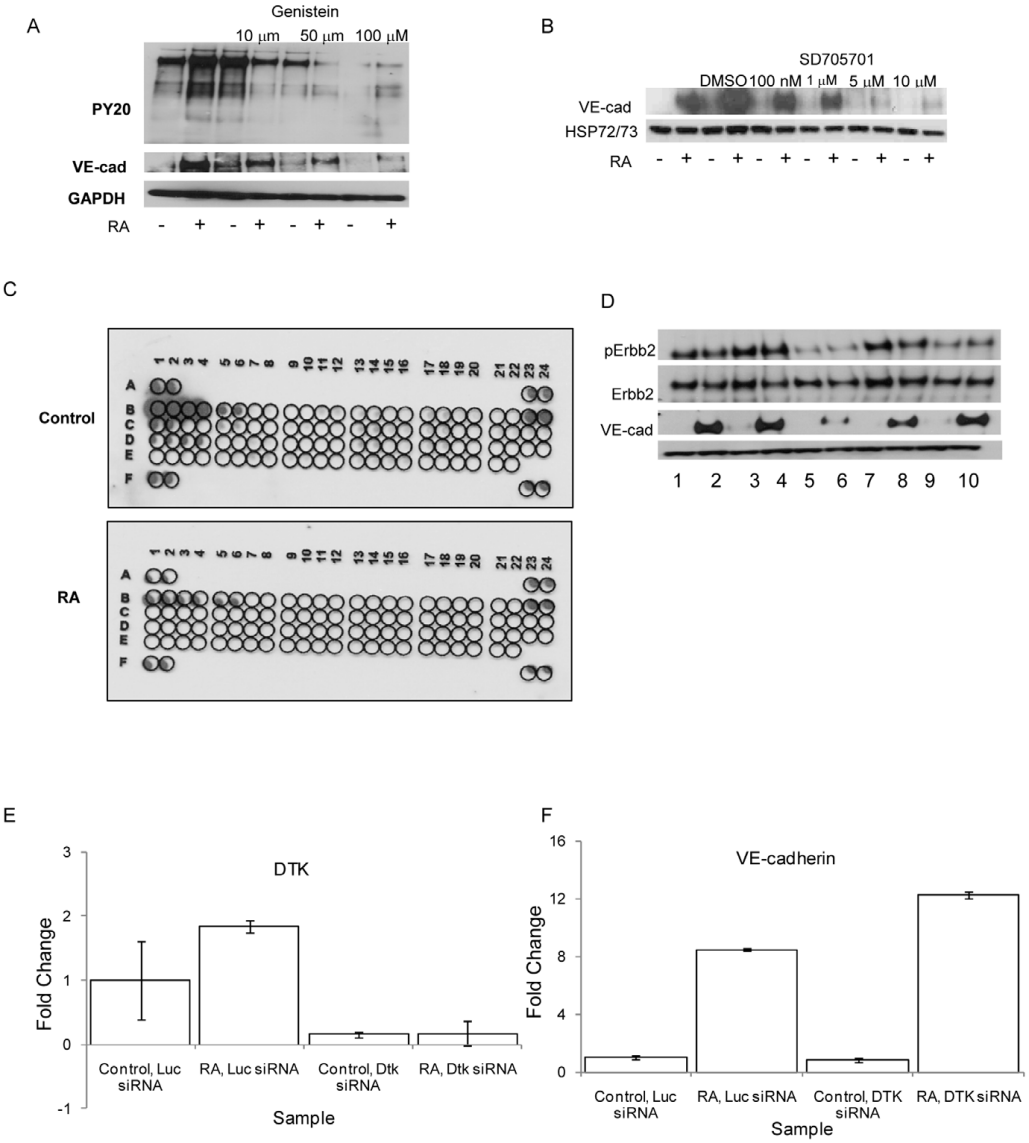
Inhibition of VE-cadherin expression occurs with treatment of pan-kinase inhibitors but is independent of tyrosine kinase activity. Pre-treatment of SKBR-3 cells for 1 hour with 10 µM, 50 µM or 100 µM of Genistein prior to a 48 hour RA treatment results in a loss of VE-cadherin expression and tyrosine phosphorylation (a). Pre-treatment with the novel pan-kinase inhibitor, SD705701, also results in a loss of VE-cadherin expression in a dose-dependent manner (b). A Human Phospho-Receptor Tyrosine Kinase array demonstrates that RA treatment results in the loss of tyrosine kinase phosphorylation (c). A key for this array is as follows: Control - A1, A2, A23, A24, F1, F2, F23, F24; EGFR - B1, B2; EphB4 - E9, E10; ErbB2 - B3, B4; ERBB3 - B5, B6; EGFR - B11, B12, B13, B14; Dtk - B23, B24; Mer - C1, C2; Tie-2 - D1, D2; TrkA - D3, D4; VEGFR2 - D11, D12. Inhibition of ERBB2 activity with AG825 is unable to inhibit VE-cadherin expression (d). Samples are labeled as follows: 1 - Control; 2 - RA; 3 - Control, Ethanol; 4 - RA, Ethanol; 5 - Control, 100 µm Genistein; 6 - RA, 100 µM Genistein; 7 - Control, DMSO; 8 - RA, DMSO; 9 - Control, 50 µM AG825; RA - 50 µM AG825. Inhibition of DTK expression with siRNA (e) is unable to inhibit VE-cadherin expression (f).

Since kinases play important roles in most steps of angiogenesis and vasculogenesis, we were interested in determining whether kinase activity is necessary for expression of VE-cadherin in RA treated SKBR-3 cells. Previous studies have indicated that Genistein plays a role in regulating VE-cadherin expression and vascular network formation in HUVECs [42]. Following an hour of pre-treatment with two pan-kinase inhibitors, Genistein (Figure 2a) and SD705701, a novel kinase inhibitor (Figure 2b), VE-cadherin expression was lost following 48 hours of RA treatment. Each experiment was conducted three times and representative blots are shown. Since Genistein and SD705701 both target tyrosine kinases we next tested the roles of receptor tyrosine kinases (RTKs) in regulating VE-cadherin expression. SKBR-3 cells were treated with control ethanol or 10^−7^ M RA for 48 hours and tyrosine kinase activity was analyzed using a Human Phospho-Receptor Tyrosine Kinase array. Tyrosine kinase phosphorylation was broadly and markedly reduced following treatment with RA (Figure 2c). Although the Tyrosine Kinase array demonstrates a decrease in tyrosine kinase activity following RA treatment, we decided to examine the role of ERBB2 in regulating VE-cadherin expression. SKBR-3 cells are known to overexpress ERBB2, and it was a candidate regulator of VE-cadherin expression. Pre-treatment of SKBR-3 cells with AG825, an ERBB2 specific inhibitor, for 1 hour prior to a 48 hour treatment with RA was unable to regulate VE-cadherin expression (Figure 2d). The only receptor tyrosine kinase whose activity appears to remain the stable following RA treatment is DTK. DTK is a member of the AXL family of receptor tyrosine kinases. Downregulation of DTK expression via DTK specific siRNA (Figure 2e) was unable to inhibit VE-cadherin mRNA expression (Figure 2f), thereby eliminating its role as a regulator of VE-cadherin. In addition to inhibiting receptor tyrosine kinases, both Genistein and SD705701 also have activity against serine/threonine kinases, including the TGFβ pathway.

### TGFβR1 activity is necessary for VE-cadherin expression, but TGFβ1 alone is unable to induce VE-cadherin expression

**Figure 3:**
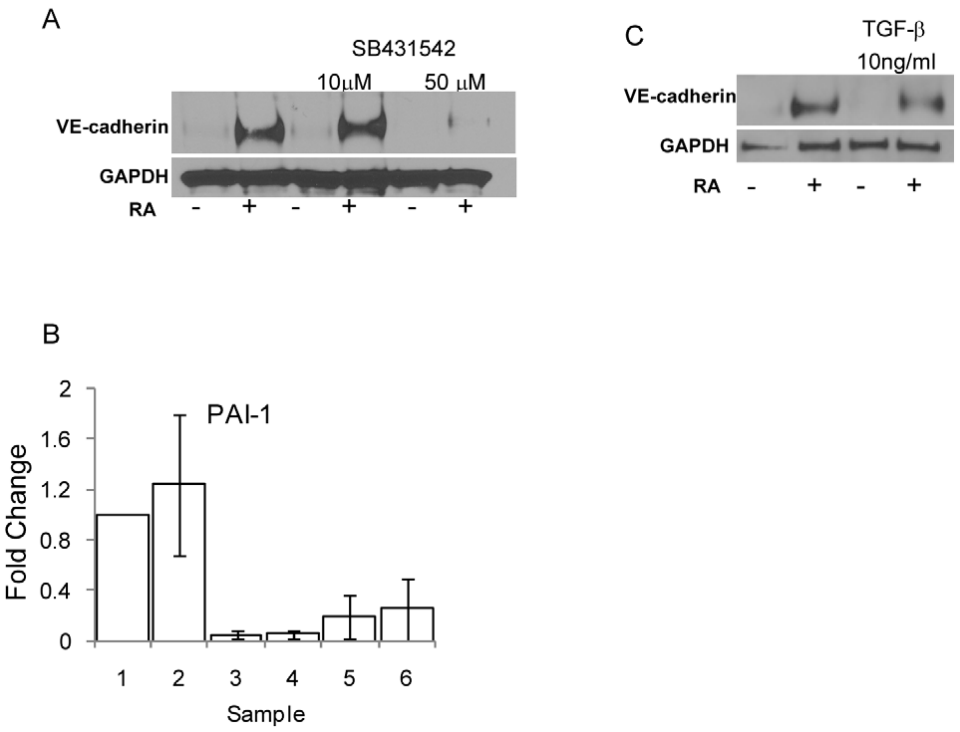
TGFβR1 kinase activity is necessary for VE-cadherin expression. A one hour pre-treatment with SB431542, a TGFβR1 kinase specific inhibitor, prior to a 48 hour RA treatment results in a loss of VE-cadherin expression in a dose-dependent manner (a). Treatment with SB431542 decreases PAI-1 activity, confirming TGFβR1 blockade (b).The samples are as follows: 1 - Control; 2 - RA; 3 - Control, 10 µM SB431542; 4 - RA, 10 µM SB431542; 5 - Control - 50 µM SB431542; 6 - RA, 50 µM SB431542. Treatment with 10 ng/ml of TGFβ, alone, does not induce VE-cadherin expression in the absence of RA treatment (c).

Ingenuity Pathway Analysis of our microarray data comparing control cells to RA treated cells indicates that several members of the TGFβ family of serine/threonine kinases were regulated by RA. These include bone morphogenic protein 7 (BMP7), SMAD1, SMAD3, transforming growth factor-β 1 (TGFβ1), and transforming growth factor-β receptor 2 (TGFβR2) (Table 3). Treatment of SKBR-3 cells with SB431542, a specific TGFβR1 kinase inhibitor, completely blocked the RA mediated expression of VE-cadherin (Figure 3a). Inhibition of TGFβR1 activity was confirmed via qPCR using PAI-1 activity as a marker (Figure 3b). However, treatment with TGFβ1, alone, is unable to induce VE-cadherin expression in the absence of RA treatment (Figure 3c). These results indicate that TGFβR1 activity is necessary but not sufficient for the RA mediated induction of VE-cadherin expression.

**Table 3:** TGFβ Family Members Regulated by RA Treatment.

Gene Name	Fold Change	p-value
TGFB2	1.9	2.8E-03
TGFB3	0.4	3.9E-05
TGFBI	2.8	1.0E-04
TGFBR1	0.5	1.6E-02
TGFBR2	2.7	2.2E-05
BMP3	1.9	1.1E-02
BMP7	0.4	6.2E-04
SMAD1	2.3	1.3E-02
SMAD3	2.4	2.1E-04
GRB2	1.9	2.5E-03

### VE-cadherin and TGFβR1 are necessary for cell fusion, but not network formation, while COUP-TFII is necessary for network formation but not cell fusion

**Figure 4:**
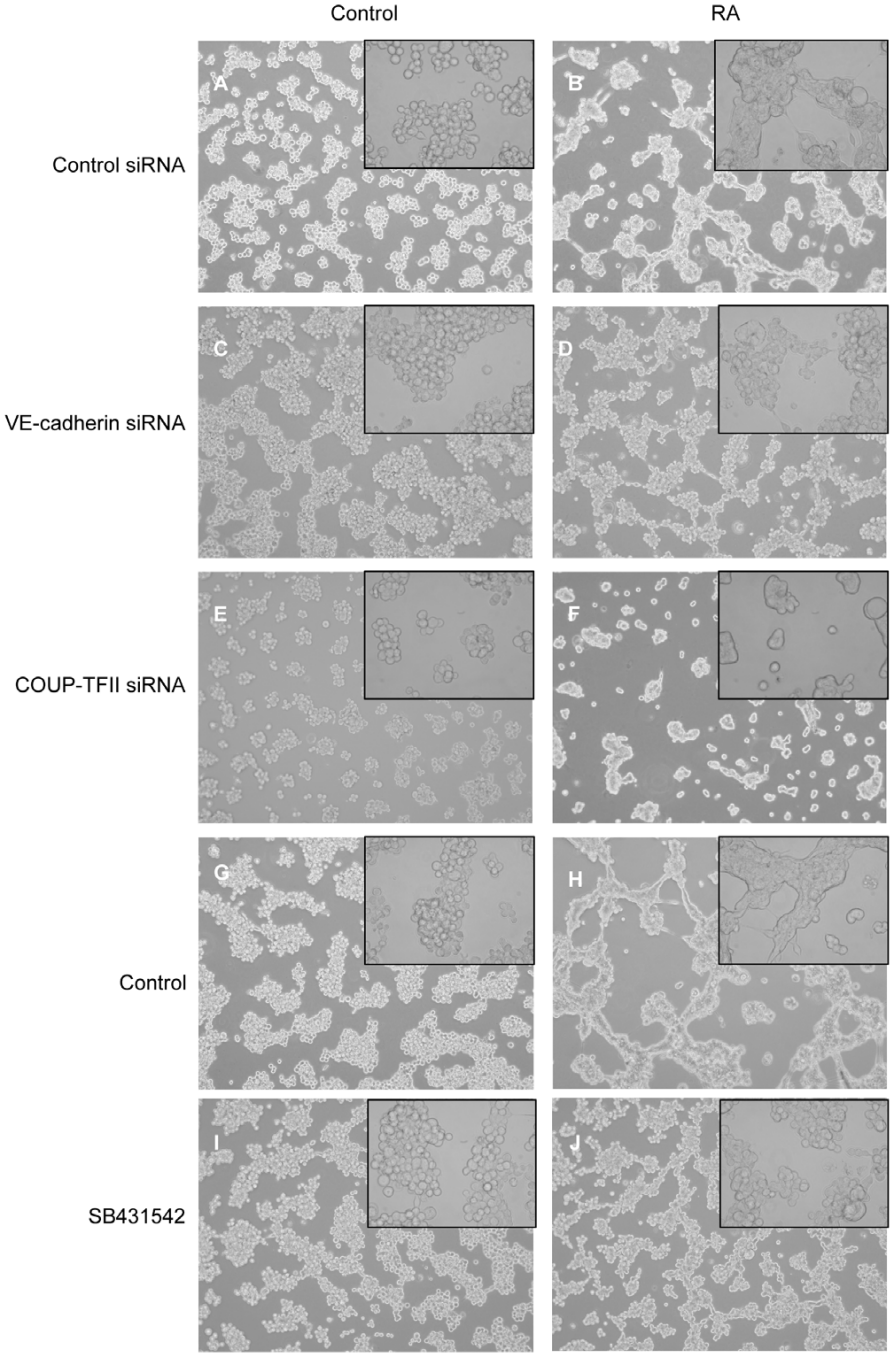
COUP-TFII is necessary for network formation while TGFβR1 activity is necessary for cell fusion. Low power images (20×) of control siRNA transfected untreated SKBR-3 cells show that they grow as clusters of individual cells in Matrigel (a) without any cell fusion (inset, 40×). Upon RA treatment, the cells begin to form networks (b) with cell fusion (inset). Loss of VE-cadherin expression using VE-cadherin siRNA does not affect untreated SKBR-3 cells (c, inset). However, a loss of VE-cadherin expression with concomitant RA treatment results in a preservation of network formation (d), but a loss of cell fusion (inset). Like the VE-cadherin siRNA treatment, COUP-TFII siRNA treatment does not affect the growth patterns of untreated SKBR-3 cells (e, inset). However, treatment with COUP-TFII siRNA does not affect the fusion of RA treated SKBR-3 cells (f, inset), but it does inhibit network formation (f). Untreated SKBR-3 cells also grow as clusters of unfused individual cells in the absence of RA treatment (g, inset), and RA treatment results in network formation and cell fusion (h, inset). Pre-treatment with 50 µM SB431542 does not affect the growth patterns of untreated SKBR-3 cells (i, inset). Pre-treatment with SB431542 prior to RA treatment does not alter network formation (j), but inhibits cell fusion (j, inset).

While neither VE-cadherin, COUP-TFII, nor TGFβR1 are master regulators of the endothelial genetic program, they may play important roles in the cell fusion and network formation that occurs following RA treatment [19]. SKBR-3 cells were transfected with control siRNA and pretreated for 24 hours with either ethanol control or 10^−7^ M RA for 24 hours prior to plating in Matrigel. When control treated SKBR-3 cells are gown in Matrigel, they grow as grape like clusters (Figure 4a) and do not fuse (inset). In the presence of RA, SKBR-3 cells form network-like structures (Figure 4b) and begin to fuse, and boundaries between cells are indistinguishable (inset). In the presence of VE-cadherin siRNA, control SKBR-3 cells grow like the control siRNA transfected SKBR-3 (Figure 4c) and do not fuse (inset); however, VE-cadherin siRNA transfected cells treated with RA still form rudimentary networks (Figure 4d), but cell fusion is greatly reduced (inset). Similarly, the treatment of SKBR-3 cells with vehicle does not result in network formation or cell fusion in the absence of RA (Figure 4g), but does result in network formation and cell fusion when treated with RA (Figure 4h). Treatment of SKBR-3 cells with the TGFβR1 inhibitor (Figure 4i), SB431542, does not alter morphology in the absence of RA. SB431542 (Figure 4j) treated SKBR-3 cells that are treated with RA do not fuse (inset) but do form networks (Figure 4j) much like RA treated VE-cadherin siRNA transfected SKBR-3 cells. These results indicate that VE-cadherin expression is necessary for cell fusion, but not the network formation phenomenon. Additionally, inhibiting TGFβR1 activity mimics the effects of a loss of VE-cadherin providing further evidence that TGFβR1is necessary for VE-cadherin expression.

When SKBR-3 cells are transfected with COUP-TFII siRNA, untreated cells grow as clusters (Figure 4e) and do not fuse (inset). On the other hand, treatment with RA results in cell fusion (Figure 4f, inset), but network formation does not occur (Figure 4f). These results indicate that COUP-TFII, which does not regulate VE-cadherin expression, is not responsible for cell fusion; however, the loss of COUP-TFII prevents the formation of networks.

## Discussion

The role of this study was to further understand the mechanism of RA mediated endothelial transdifferentiation in SKBR-3 breast cancer cells. We have previously shown that RA induces the expression of genes associated with the endothelial lineage and allows for interaction of RA treated SKBR-3 cells with HUVEC cells in Matrigel. We have shown that ER81 and SOX9 are necessary but not sufficient for inducing VE-cadherin expression. In this study, we have shown that a physiologic dose of RA regulates many genes. Some of the affected pathways are the cancer growth regulation pathway, cardiovascular development, and the hematological and coagulation pathways. Genes associated with proliferation and cancer are decreased, as is expected with RA treatment [43], [44]. As we have previously shown, genes associated with the endothelial lineage are induced [19]. These results may help explain the mixed results of RA treatment in clinical trials. In individuals with specific molecular profiles, RA treatment, while decreasing cell growth, may also push cells along deleterious differentiation pathways, such as the endothelial pathway. This differentiation may promote vasculogenic mimicry, and thus, an alternative mechanism of tumor vascularization. We utilized several markers of endothelial transdifferentiation – VE-cadherin, COUP-TFII, NRP1, EfnB2, TFPI2, and COX1 - and analyzed the roles of VE-cadherin, COUP-TFII, and NRP1 as master regulators of vasculogenic mimicry. Knockdown of VE-cadherin expression using siRNA was unable to inhibit the expression of endothelial genes when analyzed using both qPCR and microarray analysis. Surprisingly, the loss of VE-cadherin seemed to promote the expression of some pro-angiogenic genes in RA treated SKBR-3 cells. However, down-regulation of VE-cadherin expression is one of the first steps of angiogenesis; thus, this may, in fact, promote further branching morphogenesis. VE-cadherin siRNA also potentiated the RA mediated increase in coagulation/hematological factors, which is in line with the current understanding of the role of VE-cadherin in the coagulation cascade [45]. The loss of VE-cadherin did not have an effect on the growth regulatory genes that were affected by RA treatment. We also used siRNA technology to knockdown COUP-TFII and NRP1 and study the expression of endothelial-related genes on the qPCR level. Neither of these genes appears to play a role in regulating the expression of endothelial specific genes.

Given the importance of kinases in various steps of vasculogenesis and angiogenesis, we wanted to know whether kinases played a role in regulating the expression of endothelial-related genes. For these studies, we used VE-cadherin as a marker for endothelial transdifferentiation. We find that treatment of SKBR-3 cells with genistein results in a loss of VE-cadherin expression, which is similar to that observed in HUVECs treated with genistein [46]. Another pan-kinase inhibitor, SD705701, had similar effects on VE-cadherin expression at much lower concentrations. To determine whether tyrosine kinases are involved in this process, we utilized a receptor tyrosine kinase array to determine the activity of kinases following RA treatment. We found that RA treatment leads to a loss of tyrosine kinase activity. These results indicate that while kinase activity is necessary for VE-cadherin expression, it is independent of receptor tyrosine kinases.

Following closer analysis of our microarray data, we identified several members of the TGF-β family of cytokines that are induced as a result of RA treatment. SD705701 is known to have activity against serine/threonine kinases, and genistein, while inducing the expression of TGF-β has also been shown to inhibit TGFβR1 mediated phosphorylation of p38 MAPK [47]. TGF-β treatment, alone, is unable to induce VE-cadherin expression; however, inhibition of TGFβR1 activity using the specific kinase inhibitor, SB431542, inhibits RA-induced VE-cadherin expression. These results imply that activity of TGFβR1 is necessary but not sufficient for VE-cadherin expression, and presumably complete endothelial transdifferentiation. Additionally, the activation of ALK1 type I receptors induces the phosphorylation of SMAD1, SMAD5, and transcription of Id1, while activation of ALK5 receptors induces the phosphorylation of SMAD2 and the transcription of PAI-1 [40]. ALK1 has been shown to stimulate endothelial proliferation and migration, while ALK5 activation inhibits these processes. Our microarray analysis indicates that Id1 expression is induced as a result of RA treatment. Thus, the ALK1 type I receptor may also play a role in RA induced endothelial transdifferentiation.

We have also shown that network formation and cell fusion are distinct processes during vasculogenic mimicry. VE-cadherin expression, as expected, plays a role in mediating the formation of cell-cell adhesions and cell fusion. Loss of VE-cadherin expression either directly by VE-cadherin siRNA or indirectly via inhibition of TGFβR1 kinase is able to inhibit cell fusion in Matrigel, but does not appear to affect the formation of primitive network-like structures. On the other hand, loss of COUP-TFII expression using siRNA is unable to inhibit cell fusion following RA treatment, but profoundly inhibits network formation. Proper vascular formation by endothelial cells requires both processes. Network formation without cell fusion may not allow for the proper conductance of nutrients to a tumor. In the same way, cell fusion without network formation will not allow the lumen-like spaces formed by RA treated SKBR-3 cells to properly connect with the host vasculature. Thus, both processes may have to occur to allow for the deleterious effects in cancer patients.

**Figure 5:**
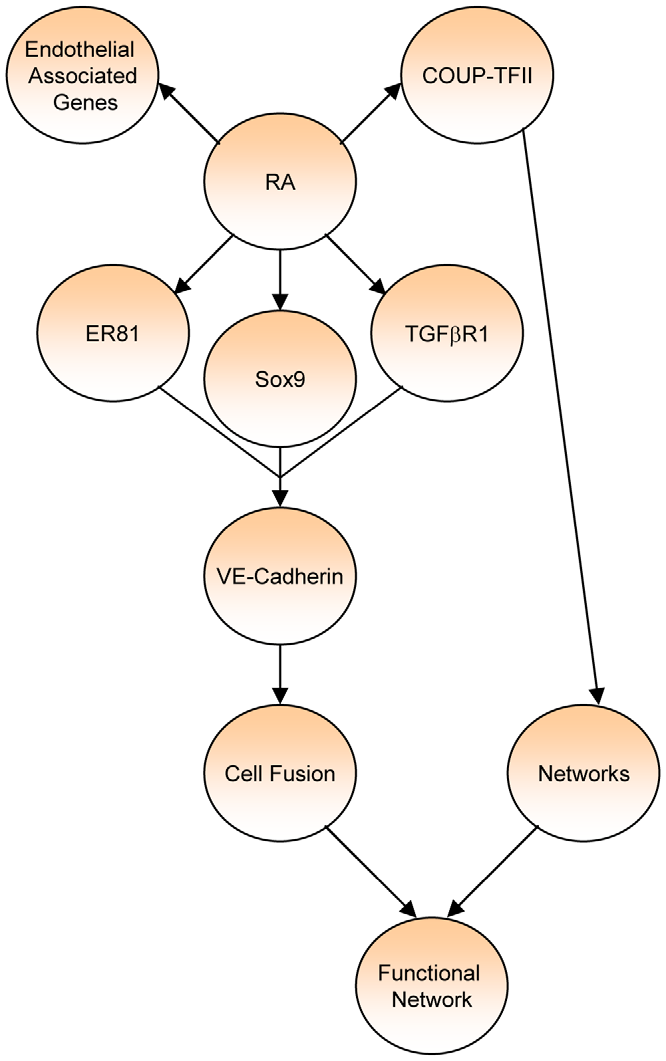
Schematic representation of factors regulating RA-mediated endothelial transdifferentiation.

The presence of vasculogenic mimicry is a negative prognosticator of patient outcomes in human cancers. We have previously identified a dietary agent, vitamin A, in regulating this process in breast cancer. We now propose that VE-cadherin expression induced by RA is regulated by ER81, SOX9, and TGFβR1 activity, while network formation is regulated by COUP-TFII (Figure 5).

## Materials and Methods

### Materials and Reagents

9-*cis*-RA was obtained from Sigma-Aldrich (Germany), mouse anti-VE-cadherin was obtained from R&D systems (Minneapolis, MN) and anti-GAPDH was from Research Diagnostics Inc (Flanders, NJ). Small interference RNA (siRNA) reagent (SMART pool) for human VE-cadherin, human COUP-TFII, and human NRP1 were purchased from Dharmacon (Lafayette, CO). As a control siRNA, Luc siRNA was used (CGUACGCGGAAUACUUCGA). We obtained TGFβ1 (100–21) from PeproTech (Rocky Hill, NJ), SB431542 from Sigma-Aldrich (Germany). Genistein was kindly provided by Dr. Partha Banerjee (Georgetown University, Washington, DC). The Human Phospho-RTK Array was performed as per the manufacturer's instructions (R&D Systems, Minneapolis, MN).

### Cell Culture and Transfection

SKBR-3 cells were obtained from the ATCC (Manassas, VA). Early passage (< passage 30) SKBR-3 cells were maintained in Dulbecco's Modified Eagle's Medium (DMEM) supplemented with 10% fetal bovine serum (FBS) in 5% CO_2_ incubator at 37°C. All transient transfection experiments of plasmid DNA and siRNA were performed with Amaxa electroporation system (Amaxa, Inc, Gaithersburg, MD) according to the manufacturer's protocol.

### Inhibitor Studies

SKBR-3 cells were plated in 6-well plates at a density of 500,000 cells per well. Cells were pre-treated with the inhibitor or vehicle for 1 hour at 37°C in a 5% CO_2_ incubator. Cells were then treated with 0.1 µm RA and incubated for an additional 48 hours. Each experiment was performed a minimum of three times.

### Matrigel Assays

For the siRNA experiments, SKBR-3 cells were transfected with the appropriate siRNA and incubated for 8 hours prior to the addition of 10^−7^ M RA. For the inhibitor experiments, SKBR-3 cells were pre-treated with inhibitor for 1 hour prior to the addition of 10^−7^ M RA. Following the addition of RA, the siRNA transfected cells and inhibitor treated cells were incubated for 24 hours at 37°C in a 5% CO_2_ incubator. The next day, each well of a 12-well glass-bottom dish (MatTek, Ashland, MA) was coated with 120 µL of Matrigel (BD Biosciences, San Jose, CA) and incubated 15 min at 37°C. SKBR-3 cells (100,000 cells/100 µl medium) were gently plated on top of the Matrigel layer directly and further incubated 30 min at 37°C. One milliliter of growth medium was added along with either ethanol control or the appropriate concentration of 9-*cis*-RA and inhibitor, if appropriate. Cells were maintained at 37°C in a 5% CO_2_ incubator. Cells were visualized using the Olympus IX71 inverted fluorescent microscope. Each study was performed a minimum of three times.

### Microarray Analysis

SKBR-3 cells were incubated in the presence or absence of 9-*cis*-RA (0.1 µM) for 48 h. Total RNA was isolated using Trizol (Invitrogen) combined with RNAeasy (Qiagen, Valencia, CA) and was amplified according to the Affymetrix protocol (GeneChip Eukaryotic Small Sample target labeling Assay Version II) modified so that the ethanol precipitation cDNA cleanup step was substituted by Qiaquick PCR purification kit (Qiagen). Biotin-11-CTP and biotin-16-UTP (Enzo Diagnostics, Farmingdale, NY) was incorporated during in vitro transcription and 20 ug of the biotinylated cRNA product was fragmented at 94°C for 25 min. Treated and untreated samples were amplified, labeled, fragmented and hybridized in the same run. Hybridizations to Affymetrix HG-U133A GeneChips were performed at 45°C for 16 h followed by staining and washing as described in the manufacturer's instructions. The processed chips were then scanned using an Affimatrix GeneArray scanner. Grid alignment and raw data generation were performed using Affymetrix GeneChip 5.0 Software. For quality control, oligo B2 was hybridized to the chip and the checkerboard pattern in each corner of the chip analyzed. BioB, bioC and bioD probes are added to each sample to standardize hybridization, staining and washing procedures. Raw expression values representing the average difference in hybridization intensity between oligonucleotides containing single base pair mismatches, was measured. The data from three independent experiments was analyzed using BRB Array tools (National Cancer Institute, Bethesda, MD).

### Network Generation

A data set containing gene identifiers and corresponding expression values was uploaded into Ingenuity Pathway Analysis. Each gene identifier was mapped to its corresponding gene object in the Ingenuity Pathways Knowledge Base. Focus genes, were overlaid onto a global molecular network developed from information contained in the Ingenuity Pathways Knowledge Base. Networks of these focus genes were then algorithmically generated based on their connectivity.

### Real-time quantitative PCR

Relative quantification was used to evaluate the raw data obtained from real-time PCR (7900 HT real time PCR system, 384 well format, Applied Biosystems, Foster City, CA). All probes were purchased from Applied Biosystems (Foster City, CA). All standards and unknowns were performed in triplicate. The average value of the triplicate readings for each unknown was then divided by the corresponding value for GAPDH RNA to normalize the data. After normalization, the value obtained for the treated unknown was divided by the value obtained for the corresponding untreated sample. The final value obtained was a measure of the fold change in gene expression for the particular genes of interest between the treated sample and the untreated sample. Each experiment was performed at least three times and the standard error of the means was calculated.

### Immunoblotting

Eighty-90% confluent SKBR-3 cells were incubated with DMEM in the presence or absence of 9-*cis*-RA for 48 hours under the indicated conditions. Cells were rinsed twice with PBS and lysed with buffer containing 1% NP-40, 1% sodium deoxycholate, 0.1 % SDS, 150 mM NaCl, 10 mM sodium phosphate, pH 7.2 and complete mini protease inhibitors (Roche Applied Science, Indianapolis, IN). Cell lysates were clarified by centrifugation at 14,000 rpm for 10 min at 4°C. Protein concentration was determined with a Bio-Rad DC reagent (Bio-Rad, Hercules, CA). After SDS-PAGE, proteins were transferred to Immobilon P (Millipore, Billerica, MA). Membranes were blocked with 5% milk in Tris-Buffered Saline containing 0.1% Tween-20, and incubated with primary antibody overnight at 4°C and subsequently with HRP-labeled secondary antibody. Proteins were visualized with ECL reagents (Amersham Biosciences, Piscataway, NJ) or SuperSignal West Femto (Pierce biotechnology Inc., Rockford, IL), using X-ray films (Denville Scientific Inc., Metuchen, NJ). Each experiment was performed a minimum of three times.

## Supporting Information

Table S1 Tumor Morphology Genes Regulated by RA.
(0.03 MB XLS)

Table S2 Cardiovascular Development and Function Genes Regulated by RA.
(0.03 MB XLS)

Table S3 Hematological Function and Development Genes + Coagulation Cascade Genes Regulated by RA.
(0.02 MB XLS)
